# There’s More to Groove than Bass in Electronic Dance Music: Why Some People Won’t Dance to Techno

**DOI:** 10.1371/journal.pone.0163938

**Published:** 2016-10-31

**Authors:** Brian C. Wesolowski, Alex Hofmann

**Affiliations:** 1 Hugh Hodgson School of Music, The University of Georgia, Athens, GA, United States of America; 2 Austrian Research Institute for Artificial Intelligence (OFAI), Freyung 6/6, A-1010, Vienna, Austria; 3 Institute of Music Acoustics, The University of Performing Arts, Anton-von-Webern-Platz 1, 1030, Vienna, Austria; University of Würzburg, GERMANY

## Abstract

The purpose of this study was to explore the relationship between audio descriptors for groove-based electronic dance music (EDM) and raters’ perceived cognitive, affective, and psychomotor responses. From 198 musical excerpts (length: 15 sec.) representing 11 subgenres of EDM, 19 low-level audio feature descriptors were extracted. A principal component analysis of the feature vectors indicated that the musical excerpts could effectively be classified using five complex measures, describing the rhythmical properties of: (a) the high-frequency band, (b) the mid-frequency band, and (c) the low-frequency band, as well as overall fluctuations in (d) dynamics, and (e) timbres. Using these five complex audio measures, four meaningful clusters of the EDM excerpts emerged with distinct musical attributes comprising music with: (a) isochronous bass and static timbres, (b) isochronous bass with fluctuating dynamics and rhythmical variations in the mid-frequency range, (c) non-isochronous bass and fluctuating timbres, and (d) non-isochronous bass with rhythmical variations in the high frequencies. Raters (*N* = 99) were each asked to respond to four musical excerpts using a four point Likert-Type scale consisting of items representing cognitive (*n* = 9), affective (*n* = 9), and psychomotor (*n* = 3) domains. Musical excerpts falling under the cluster of “non-isochronous bass with rhythmical variations in the high frequencies” demonstrated the overall highest composite scores as evaluated by the raters. Musical samples falling under the cluster of “isochronous bass with static timbres” demonstrated the overall lowest composite scores as evaluated by the raters. Moreover, music preference was shown to significantly affect the systematic patterning of raters’ responses for those with a musical preference for “contemporary” music, “sophisticated” music, and “intense” music.

## Introduction

Operational definitions of groove include “a pleasant sense of wanting to move with music” [1] and “a quality of music that makes people tap their feet, rock their head, and get up and dance” [2]. However, sensorimotor coupling is not a necessary component to the experience of groove [3]. Groove can also be described as one’s ability to simply derive a satisfaction and enjoyment through the understanding and appreciation of the rhythmic aesthetic of a particular style of music [4]. The act of listening to music, without movement, can therefore result in an experience of groove [1]. Human responses to groove-based music, therefore, can be categorized into three distinct dimensions: (a) formal-syntactic-intellectual (i.e., cognitive response), (b) expressive-emotional (i.e., affective response), and (c) and embodied response (i.e., psychomotor response) [5–7]. In this study, we explored the relationship between raters’ perceived cognitive, affective, and psychomotor responses and the audio descriptors for groove-based music. In particular, we sought to investigate electronic dance music, a music specifically composed for evoking dance movement.

### Cognitive Response

From a cognitive perspective, Toussaint [8] indicates that, “… rhythm perception emerges from the interplay between bottom-up, data-driven, outer stimuli emanating from the world, and the top-down, conceptually-driven, inner response mechanisms of the mind’s ear” (p. 10). Eliciting meaning from multisensory input, however, is a contextually driven, top-down sensory process requiring a system of complex auditory abilities to extract, organize, and interpret acoustic information [9]. Cognitive interpretation and recognition of rhythmic structure in groove-based music is an interaction between two schemes [10]. The *first scheme* includes the personal and subjective representation to non-sounding “virtual reference structures,” whereby a listener has identified then internalized the rhythmic syntax of a given musical idiom [3]. These virtual reference structures include perceptually driven, organizing principles such as (a) formal units (e.g., motives and phrases); (b) temporal units of hierarchically nested intervallic timing systems (e.g, beats and subdivisions), and (c) surface features (e.g., harmonic, melodic, and rhythmic tension) [11,12]. Structural meaning in groove-based music is elicited by the listener through the identification of idiomatic variations within each of these rhythmic structures. More specifically, this includes the subtle deviations of four particular expressive dimensions of sound (i.e., auditory cues): (a) intensity, (b) intonation, (c) articulation, and (d) timbre [4,11]. The *second scheme* includes the personal and subjective representation to actual acoustic events. In the listening experience, acoustical events related to rhythmic structure are used to make a perceptual judgment that is associated with the categorical/structural component [13].

### Affective Response

Psychological investigation into affective behavior includes the evaluation of dimensions of feeling aroused by a stimulus. Specifically, affect can be defined as a broad term applied to a wide variety of human feeling behaviors as well as the type or level of arousal resulting from a musical listening experience [14]. In groove-based music, the nature of how one perceives groove as being good or bad, groovy or not groovy, is based upon subjective reception [3]. Positive affect in groove music may be based upon a “reward system,” that “arises from interactions between cortical loops that enable predictions and expectancies to emerge from sound patterns and subcortical systems responsible for reward and valuation.” [15] (p. 1). Momentary violations of temporal expectancy evoke arousal through delayed gratification [16–19]. More specifically, the relationship between rhythmic complexity and valuation of groove is in the form of an inverted U-shaped curve, indicating that positive affect is optimized when the music involves an intermediate degree of rhythmic complexity [7].

The ability to move in rhythmic synchrony to an isochronous musical pulse is a universal and relatively unique human quality [20,21]. The theory of embodied cognition supports the notion that auditory motor coupling emerges from real-time interactions between the body’s nervous system as well as perceptions of events. Action and perception are intertwined in the music listening experience and share a common representational domain [22]. The parity between perception and action is supported in common coding theory [23] under two assumptions: (a) actions are coded in terms of the perceivable events [24]; and (b) the perception of an action should activate action representations to the degree that the perceived and the represented action are similar [25]. Multisensory input (i.e., cognitive and affective information) are guided by motor images of the bodily experience, where cognitive structures can emerge from recurrent sensorimotor patterns. Therefore, cognitive and affective information is an important component to the physical engagement of a music listening experience in groove-based contexts.

### Psychomotor Response

A positive drive toward sensorimotor coupling is an important factor in how listeners emotionally engage with and enjoy groove-based music. This plays a prominent role in evaluating listeners’ experiences and the strength of engagement with groove-based musical stimuli [26]. Pressing [27] refers to the physical response to groove-based music as a “kinetic framework for reliable prediction of events and time pattern communication…” where “… its power is cemented by repetition” (p. 306). According to Zbikowski [28]:

Listeners who respond physically to a groove have succeeded in selecting salient features out of a sequence of sounds and related these features in such a manner that they were able to identify a sense of regularity, differentiation, and cyclicity in the music. (p. 279)

Organizing principles of rhythmic structure are based upon cognitive interpretation, whereas enjoyment of groove-based music is based upon affective interpretation. As a consequence, the sense of wanting to move to groove-based music is a result of interplay between cognitive and affective interpretations. Therefore, the study of cognitive, affective, and psychomotor responses to groove-based music requires a multi-leveled attention to human perception [3].

### Electronic Dance Music (EDM)

The advent of electronic dance music (EDM) began in the 1980s, when music producers and composers started using digital sequencers and electronic instruments for the production of groove-based music. This enabled them to produce quantized grooves with a higher temporal precision than human drummers could play. This new aesthetic lead to the origin of the electronic dance music (EDM) (see Butler [29] for an overview). It became popular for DJs to create remixed dance versions of traditional rock and pop music by sampling the original tracks, aligning these to the time grid of a software sequencer and to add quantized beats. In these instances, reinterpretations of the rhythmic groove pertained to variations and layering of metrical patterns, accents, and timbres, thereby providing new avenues for many subgenre classifications [30,31].

Berger [32] suggests that in groove-based music, “Each [musical] style has a vocabulary of grooves, based upon a variance of patterns of notes and accents under a specified hierarchy of perceived strong and weak beats within a time signature framework.” Butler [29] provides a specific classification system for EDM based on two rhythmic characteristics: (a) the syncopated 'breakbeat' styles, and (b) the 'four-on-the-floor' styles. 'Breakbeats' are sampled drum patterns of real percussion instruments mostly stemming from funk music of the 1970s. These ‘breakbeats’ are characterized by syncopations that de-emphasize the strong beats and their deviations from total isochrony. In contrast, 'Four-on-the-floor' genres are based on programmed drum machine beats that emphasize the strong 4/4 beats with the bass drum sound.

Although EDM can be broadly described as having a repetitive beat based on a 4/4 time-grid and simple phrase lengths of 2, 4, 8, or 16 bars, hundreds of subtle subgenres exist [30]. These subgenres have characteristic aesthetics containing both similarities and differences in their metrical patterns, accents, and timbres. It is argued that the subgenre labels of EDM are not concretely defined, thereby providing an unclear representation of structural cues based upon genre labeling [30,31,33]. Therefore, in order to evaluate human response to various subgenres of EDM, it is necessary to first develop a more clear understanding of the specific audio signal properties of the wide variety of EDM subgenres.

Gjerdingen and Perrott [34] provided evidence that listeners were able to categorize music samples into genres within a quarter of a second. From these observations, they hypothesized that this rapid recognition of the genre is based on decoding component features of the sound instead of a classification of melody, harmony and rhythm. They acknowledge that a discrimination of sub-genres is a more difficult task, but individuals who are familiar with a preferred genre are more likely to find subtle distinctions than others (i.e., "Fisheye-Lens Effect").

Rocha, Bogaards, and Honingh [35] used *timbre features* to find similarities and differences between EDM songs in a large music database. They created a feature vector comprising properties of the spectral envelope, the spectral flatness and the spectral roughness. Based on this feature vector, they created a similarity map of the audio clips. In a follow-up report published on their project web-site, they also added rhythmic similarity to their feature vector to gain better discriminatory results [36].

Madison, Gouyon, Ullén, and Hörnströ [37] investigated in how far low-level audio descriptors can predict groove in music clips of five different genres (e.g., Greek, Indian, Jazz, Samba, West African). Out of five descriptors (beat salience, fast metrical levels, event density, systematic microtiming, and unsystematic microtiming) they found two descriptors function: a) *beat salience*, a measure for the repetitive rhythmical patterning; and b) *event density*, a measure for the density of sound events. They reported that rhythmical descriptors played a larger role in predicting groove than the systematic and unsystematic microtiming descriptors that have been considered to create a sensation of groove for many years. In our study, where the pool of stimuli consists of repetitive, quantized, electronic groove music, basic low level audio descriptors like *timbre* and *event density* are considered to be useful tools to characterize the stimuli.

The overall purpose of this study is to explore the relationship between audio signal properties of electronic dance music (EDM) and raters’ perceived cognitive, affective, and psychomotor responses. Specifically, this study was guided by the following research questions:

1. Which low level audio descriptors (LLD) meaningfully classify the musical excerpts taken from different electronic dance music subgenres?
2. Does a meaningful typology of the musical excerpts of electronic dance music (EDM) exist based upon their audio signals?
3. How do rating scale items representing cognitive, affective, and psychomotor domains vary for the musical stimuli?
4. Do meaningful response patterns emerge for groups of raters based upon musical preference?

### Psychometric Considerations for Rater Responses

The measurement of music perception and cognition with raters is fraught with difficulties, as non-observable phenomena are the object of measurement [38]. Therefore, as Keller and Schubert [5] note, cognitive and affective research literature related to raters’ enjoyment of music, moderated by perceived emotional content and complexity, are “not always consistent” (p. 144). When using rater data as a method for quantifying objects of measurement such as musical stimuli, ratings are associated with characteristics of the raters and not necessarily with the musical samples themselves [39]. Therefore, threats to construct-irrelevant variability exist that may obscure what is intended to be measured, the use of the measurement apparatus itself, and the resulting observed scores [40,41]. Developments in modern measurement theory offer improved methods for systematically measuring attitudes and perceptions mediated by raters in meaningful and objective ways [42].

Rasch measurement theory [43] was used in this study for the psychometric evaluation of rater data and audio features. Rasch models use probabilistic distributions of responses as a logistic function of person (or in this specific context, a musical stimulus) and item parameters in order to define a unidimensional latent trait. The understanding and detection of unidimensionality in the context of this study is of importance. In this study, we were interested in measuring raters’ responses to musical stimuli. A multidimensional approach that examines and interprets various interaction effects may potentially skew the interpretation of the data. The Rasch measurement model was specifically chosen as a suitable method for analysis as it is grounded in principles of fundamental measurement that emphasizes the measurement of one dimension at a time [44–46].

The major benefit of the Rasch model is that when adequate fit to the model is observed, five requirements for rater-invariant measurement are achieved [40]. The five requirements for rater-invariant measurement include: (a) rater-invariant measurement of persons (i.e., *the measurement of musical stimuli must be independent of the particular raters that happen to be used for the measuring*); (b) non-crossing person response functions (i.e., *higher qualifying musical stimuli must always have a better chance of obtaining higher ratings from raters than lower qualifying stimuli)*; (c) person-invariant calibration of raters (i.e., *the calibration of the raters must be independent of the particular musical stimulus used for calibration*); (d) non-crossing rater response functions (i.e., *any musical stimulus must have a better chance of obtaining a higher rating from lenient raters than from more severe raters)*; and (e) musical stimuli and raters must be simultaneously located on a single underlying dimension. When the data fit the requirements of the Rasch model, then it becomes possible to support rater-invariant measurement of the musical stimuli. In other words, construct-irrelevant factors such as the unique characteristics of musical stimuli, raters, or rating scale items do not contribute any interference between observed data and expectations of the Rasch model. Therefore, the Rasch measurement model approach is considered a powerful tool for providing a comprehensive view of the validity of raters’ scores, the meaning of the scores, and the scores’ utility through the use of a rigorous set of measurement requirements. This method provides a valid mechanism to explore the relationship between the audio signal properties of electronic dance music (EDM) and raters’ perceived cognitive, affective, and psychomotor responses.

## Method

### Participants

Raters (*N* = 99; *n* = 63, male; *n* = 36, female) were non-music university students from a large southern state university in the United States. All of the participants had experience playing musical instruments in a concert band setting. The average age of participants was 20.68 (range 18–24). Participants were solicited on a volunteer basis and did not receive any reward or payment for their participation.

### Stimuli

Electronic dance music samples (*N* = 198) were gleaned from Beatport.com’s Top 100 charts of the year 2014 representing each the following subgenres: Breakbeats (*n* = 18), Chillout (*n* = 18), Deep House (*n* = 18), Drum and Bass (*n* = 18), Dubstep (*n* = 18), Electro House (*n* = 18), House (*n* = 18), Progressive House (*n* = 18), Tech House (*n* = 18), Techno (*n* = 18), Trance (*n* = 18). The complete list of musical stimuli can be found in S1 Table.

Musical samples were examined for sections that contained only a repetitive groove in order to avoid the influence of ratings via changes in rhythm, other auditory cues, or lyrics [47–50]. Each section was parsed into a sixteen-measure (approximately 15 seconds) sample, containing no breaks or lyrics, but representative of the groove. The audio files were normalized in volume and edited to include a three second fade in and a three second fade out using Logic Pro X (by Apple Inc.).

### Rater Evaluation Plan

The structure of how the raters evaluated the musical stimuli was carefully constructed based upon an incomplete assessment network as recommended by Wright and Stone [51] and Linacre and Wright [52]. According to Linacre and Wright, implementation of the collected data:

… does not require that every examinee [in this case, musical sample] be rated by every judge on every item. It is only necessary that observations are designed to create a network through which every parameter can be linked to every other parameter, directly or indirectly, by some connecting observations. The network enables all measures and calibrations estimated from the observations to be placed on one common scale.” (Linacre & Wright, 2004, p. 300).

In this study, each rater evaluated four random musical stimuli, chosen at random with the constraint that the stimuli came from four different subgenres. From the four musical samples, two samples were linked to the previous rater and two samples were linked to the next rater. As an example, Rater 01 evaluated samples 001, 002, 003, 004; Rater 02 evaluated samples 003, 004, 005, 006; Rater 03 evaluated samples 005, 006, 007, 008, etc. The last rater (i.e., Rater 99) evaluated musical samples 197, 198, 001, and 002, thereby linking to Rater 01 and creating a connection between all other raters in the model. This connectivity allowed for independent calibrations of all musical stimuli, rating scale items, and raters, and allowing each to be compared unambiguously [53]. Each musical stimulus within each subgenre was nominated for use in the study using a separate random number generator for each genre.

### Apparatus

To measure cognitive, affective and psychomotor responses, two measurement instruments were chosen and extended. First, Bartel’s Cognitive-Affective Response Test (CART-M) [54] was used as a measurement instrument. Bartel’s CART-M was chosen suitable for this study as the construct the instrument purports to measure paralleled this study: cognitive/affective response to music. The instrument consists of 18 items representing two dimensions of response: cognitive (items, *n* = 9) and affective (items, *n* = 9). Bartel developed the instrument through a series of three instrument validation studies using a factor analytic approach to scale construction. The final form of the instrument was developed in Bartel’s third validation study using six replications, explaining 49.89% of the variance in ratings for the utilized sample of raters and musical samples with internal consistency (Cronbach’s Alpha) calculated at .80 for the cognitive dimension and .93 for the affective dimension (See S2 Table). The original CART-M response set was a 7-point semantic differential. In this study, the response set was changed to a 4-point Likert scale, with options including “Strongly Agree,” “Agree,” “Disagree,” and “Strongly Disagree” in order to better represent category counts, average measures, thresholds, and category fit statistics. Each rater was provided the two words from the semantic differential in order to contextualize the meaning of the item. Raters were then asked to respond solely to the positive word (i.e., “This musical sample is structured” for Item C1, unstructured-structured; “This musical sample is joyful” for Item A8, sad-joyful). The positive words were selected in order to maintain all positive directionality, as negatively worded items often demonstrate a response bias and a mix of positive and negative words often demonstrate inattention and confusion [55]. A four-point scale was specifically chosen in order to eliminate neutral response options and provide a better measure of intensity of participants’ responses [51], to provide a better chance of rater reliability [56], and to optimize rating scale category effectiveness through better distribution and use of the four Likert response options [56–59]. Each volunteer rater completed the 18 items of the CART-M for each musical sample.

In order to measure psychomotor effects of the musical stimuli on the participants, participants were additionally asked to respond to the following three statements using the same 4-point Likert scale: (a) “This music makes me want to tap my feet,” (b) “This music makes me want to nod my head”, and (c) “This musical sample makes me want to dance.” These three items were added to the survey based upon Burger, et. al’s [60] investigation into music induced movement, indicating that whole body movement, feet tapping, and head movement play a significant role in the embodiment of groove-based music.

Music preference has been linked to significant differences in cognitive styles [61], personality [62] and emotion [63]. Therefore, it was advantageous in this study to consider music preference as an important moderator variable and to collect music preference information from the participants. The second apparatus used was the five-factor MUSIC model [63,64]. Participants were asked to rank order their musical preference (1, favorite -5, least favorite) based upon the five musical dimensions of the MUSIC model: (a) mellow, (b) unpretentious, (c) sophisticated, (d) intense, and (e) contemporary. Participants were provided subgenre descriptions and examples for each musical dimension in order to more clearly understand the labeling system (e.g. intense = classic rock, punk, heavy metal, power pop). For the purpose of this study, participants were categorized by their favorite musical dimension of the MUSIC model (See S3 Table).

### Procedure

#### Rater Evaluation of the Stimuli

Participants were each tested sitting at a desktop computer (iMac, by Apple Inc.) with headphones (MDR-7506, by Sony Inc.) on. the iTunes (12.0.1.26) Music playback software was used to present the stimuli to the raters. Sound was checked prior to beginning for equitable volume and equalizer settings across each computer. Using an online questionnaire (Google Forms, by Google Inc.), participants were first asked to read ethics approval information and enter background information (e.g., age and gender). Once completing the initial responses, participants were provided an electronic rater folder with four musical samples (audio files in.mp3 format) in the iTunes playback software.

Participants were instructed to listen to the first sample as many times as needed before and while responding to the items in the online questionnaire. Once completing the items for the first stimulus, they were instructed to move on to the second stimulus and continued to process through all four musical samples. The author stayed in the room and was available to address any technical problems. All participants participated with written consent through the online survey system. The University of Georgia Institutional Review Board of Human Subjects specifically approved this study.

#### Audio Analysis of Stimuli

To extract low level audio features of the stimuli used in the listening experiment, we followed a procedure similar to that of Rocha et al. [35] Audio files were down sampled to 11025 Hz to reduce the size of the data, fade-ins and fade-outs were removed. We calculated the Root Mean Square Energy of the signal (RMS), using Sonic Visualizer 1.9 (BBC vamp plug-ins). This function creates a vector describing the energy for each audio frame comprising 1024 audio samples and a reference to the actual time in the audio file. From this vector we calculated the average loudness of each stimulus (a = rmsMean). Furthermore, we calculated the standard deviation of all RMS values from the audio frames of each stimulus (b = rmsSD) to provide a measure for the dynamical fluctuations in the sound.

To characterize the timbre of each stimuli we calculated two measures: (1) the Spectral Centroid, as the center of the spectral energy [65]; and (2) the Spectral Flux, a measure for the amount of spectral fluctuations over time (Sonic Visualizer, BBC vamp plug-ins). From these time series, we calculated the arithmetic mean (c = centMean, d = fluxMean) and the standard deviation (e = centSD, f = fluxSD) to use as a descriptor for each stimulus. As an example, a low bass note (80 Hz) with a following punchy snare drum (with overtones up to 5000 Hz) would result in a higher centMean (= 990 Hz) and fluxMean (= 422 Hz), than just a bassline with sine tones of 80 Hz and 120 Hz centMean (= 106 Hz), and fluxMean (= 92).

Another descriptor, also introduced by Rocha [36], describes the auditory roughness (dirtiness) of the sounds in an audio sample. We used the roughness (g) function by Vassilakis from the Matlab MIRtoolbox [66] to extract this feature from our stimuli.

Following the methodology of Rocha [36], we calculated rhythmical descriptors for different frequency bands by splitting the spectrum of the audio samples into four frequency bands: Low (20–200 Hz), Mid1 (200–800 Hz), Mid2 (800–3200 Hz) and Hi (3200–5512.5Hz, the nyquist frequency of the audio clips). We extracted note-onsets for all four frequency bands by applying an onset-detection algorithm [67] available as a plugin for Sonic Visualizer. From these note-onsets, we calculated an Event Density measure, following the basic concept of calculating the ratio of onsets per time unit (here in seconds) for each band (h = evDensityLow, i = evDensityMid1, j = evDensityMid2, k = evDensityHi).

From the tempo information of each track, we were able to calculate the metrical level of the note-onsets of each frequency band by (evDensityMetrLevel _(l–m)_ = Event Density _(h, i, j, k)_ * 60 / Tempo). Here, a value of 1 corresponds to note-events at the quarter note level, values less than 1 indicate lower metrical levels, and values greater than 1 indicate higher metrical levels. For example, this descriptor could indicate a four-on-the-floor bass drum, by giving a value of 1 for the Low frequency band note onsets (evDensityMetrLevelLow = 1).

Lastly, we calculated the inter-onset intervals of consecutive onsets for each frequency band by using the derivative of the onset time series. The standard deviation (SD) of the IOI sequence describes the regularity of the onsets for each frequency band (IOI-SD_(p-s)_). An IOI-SD close to zero corresponds to very regular events, whereas larger values indicate irregular events. In total we extracted 19 low-level audio descriptors to characterize each stimulus.

#### Factor Analysis of Low Level Audio Descriptors

To remove redundancy or duplication from the set of 19 audio descriptors, a principal components factor analysis was conducted. Defining the characteristics of the audio descriptors through latent factor groupings managed multicollinearity between the individual audio descriptors [68]. Additionally, by describing components of the stimuli more broadly based upon the latent factors, a better independent representation of structural properties of the music emerged. These broad groupings of structural properties allowed us to gain better insight into their roles in rater judgment.

#### Clustering of Musical Stimuli

Cluster analyses is a data mining technique that assigns cases to a set of groups so that the cases within each group are similar and those clustered into other groups are dissimilar. Cluster analysis was first used in order to explore the patterns (i.e., Euclidian distances) between the stimuli based upon their factor scores resulting from the factor analysis. Hierarchical clustering is fruitful as an initial exploratory summary method because it only requires a measure of similarity between groups of data points. Hierarchical clustering was first used as an exploratory approach to investigate how the audio signal analyses grouped together to define the musical excerpts. A non-hierarchical k-means cluster analysis was then conducted as a method for forming the most meaningful interpretation of clusters from the hierarchical cluster analysis. Specifically, the non-hierarchical clustering technique optimizes the results of the hierarchical clustering technique using the dissimilarity measure as a starting criterion.

## Results

### Research Question 1: Which Low Level Audio Descriptors Meaningfully Classify the Musical Excerpts Taken from Different Electronic Dance Music Subgenres?

A principal components factor analysis was conducted in *R statistics software* using the psych package in order to explore the relationships and dimensionality between the 19 low-level audio descriptors. In order to test for the factorability of the data, several assumptions were checked: (a) all communalities were above .30 [69]; (b) audio features with inter-item correlations which did not meet the criteria to be between .30 and .80 were removed [70] (e.g., evDensityMetrLevelMid1, evDensityMetrLevelMid2, evDensityMetrLevelHi), allowing 16 audio features to remain in the vector for further calculations; (c) the Kaiser-Meyer Olkin sampling adequacy measure (.82) was above the recommended value of .50; [70] (d) Bartlett’s test of sphericity was significant (χ^2^ = 5812.92, *p* < .001) [70]; and (e) the subject-to-variable ratio (i.e., music samples to audio features) was 198:19, above the recommended minimum ratio of 3:1 [70]. A principal component analysis with oblimin oblique rotations based upon Eigenvalues above 1.00 and an examination of a scree plot indicated a five-factor solution that explained 79% of the variance.

The result indicating a five-factor solution is provided in Table 1. The five distinct audio meta descriptor measures were labeled as follows: (I) high-frequency band rhythmical descriptors, (II) low-frequency band rhythmical descriptors, (III) dynamic descriptors, (IV) mid-frequency band rhythmical descriptors, and (V) timbre descriptors (referring to the low-level audio descriptors comprised in each factor). Using the five audio meta descriptor factors, factor scores were calculated for each of the 198 stimuli.

**Table 1:** Pattern Factor Loading, Communalities, Variance, and Factor Correlations Based Upon a Principal Components Analysis (PCA) Factoring with Oblim Oblique Rotations for 16 Audio Feature Variables. Grey areas indicate the low-level audio descriptors that characterize the respective factor.

	Factor					
	I	II	III	IV	V	
Low-level Audio Descriptors	High Frequency Band Rhythmical Descriptors	Low Frequency Band Rhythmical Descriptors	Dynamic Descriptors	Mid Frequency Band Rhythmical Descriptors	Timbre Descriptors	*h*^*2*^
IOIMid2SD	0.97	0.02	0.05	0.14	-0.04	0.79
IOIHiSD	0.84	0.00	0.02	-0.15	-0.07	0.83
evDensityHi	-0.65	0.05	0.21	0.20	-0.08	0.79
evDensityMid2	-0.56	0.02	0.18	0.34	-0.05	0.78
evDensityLow	0.07	0.90	-0.12	0.08	0.09	0.81
evDensMetrLevelLow	0.07	0.84	0.04	0.06	0.09	0.72
IOILowSD	0.10	-0.83	-0.10	0.18	0.16	0.74
rmsMean	-0.04	0.06	-0.93	0.18	0.04	0.78
rmsSD	-0.13	0.12	0.78	0.10	-0.18	0.85
centSD	0.03	0.01	0.59	0.30	0.47	0.71
IOIMid1SD	0.02	-0.04	0.07	-0.90	0.09	0.84
evDensityMid1	-0.09	0.04	-0.01	0.87	-0.05	0.87
fluxMean	-0.14	0.05	-0.12	-0.12	0.92	0.87
centMean	0.39	-0.16	0.10	-0.13	0.65	0.80
roughnessMeanFull	0.21	-0.05	-0.32	-0.35	0.49	0.81
fluxSD	-0.23	0.00	0.39	0.28	0.47	0.62
Percentage of total variance accounted for	0.25	0.18	0.19	0.21	0.17	
Cumulative variance accounted for	0.19	0.34	0.49	0.66	0.79	
*Factor intercorrelations*						
I	1.000					
II	-0.21	1.000				
III	-0.27	0.05	1.000			
IV	-0.51	0.27	0.35	1.000		
V	0.14	-0.13	-0.03	-0.15	1.000	

### Research Question 2: Does a Meaningful Typology of the Musical Excerpts of EDM Exist Based upon Their Audio Signals?

**Fig 1:**
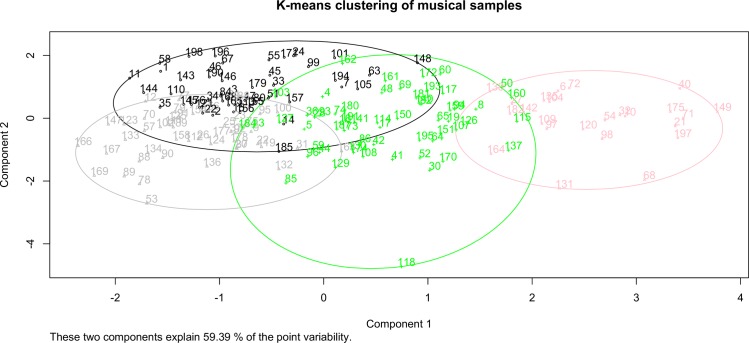
Results of K-means clustering of the stimuli for musical samples. (A) Results are derived from the five factor scores gained from the low-level audio descriptors (see Table 1). Stimuli are labeled with their Track IDs and plotted in a 2-dimensional representation (Component/Factor 1 and 2) of the four clusters (1 = black, 2 = grey, 3 = green, 4 = pink) presented in Table 2.

A hierarchical cluster analysis was conducted in *R statistics software* (hclust function) using the five derived factors for each of the 198 musical samples. Squared Euclidian distances served to maximize intra-class similarity and minimize interclass similarity. Using Ward’s method, a total of nine clusters were gleaned from the analysis. In order to target an appropriate number of clusters, Mardia, Kent, and Bobby’s “rule of thumb” [71] was considered, where the number of clusters (*k*) is approximately equal to the square root of *n* divided by *k*. Additionally, Thorndike’s elbow method [72] was considered. Lastly, the discernable and reasonable nature of substantive trends within the nine clusters was considered. Cluster solutions ranging from 2–12 were examined. Based upon the best substantive interpretation, a four-cluster solution was chosen. Four clusters were then successfully formed and validated using a k-means non-hierarchical clustering method based upon the seeds derived from the hierarchical clustering analysis (see Fig 1).

Table 2 provides the final four cluster centers based upon the five audio meta-descriptor factors. The four clusters were labeled based upon the weights of the five factors. Characteristic of the first cluster and the second cluster is a strong influence of the ‘low frequency band rhythmical descriptors’ (factor II). This factor comprises audio features describing the event density in the low frequencies (factor load ‘evDensityLow’ = 0.9) comprising primarily regular events (factor load ‘IOILowSD’ = -0.83). Both clusters (1 and 2) have different weightings for the ‘Timbre descriptors’ factor V, whereas a negative weighting indicates a more static timbre for cluster 1 (-1.18) then for cluster 2 (0.2). Furthermore cluster 2 is characterized by the strongest weight of the ‘Mid frequency band rhythmical descriptors’ factor IV (0.69).

**Table 2:** Final Four Cluster Centers by Five Factors comprising 19 low-level audio descriptors.

	I	II	III	IV	V
Cluster	High Frequency Band Rhythmical Descriptors	Low Frequency Band Rhythmical Descriptors	Dynamic Descriptors	Mid Frequency Band Rhythmical Descriptors	Timbre Descriptors
Cluster 1: Isochronous Bass / Static Timbre	-0.38	0.31	-0.14	0.38	-1.18
Cluster 2: Isochronous Bass / Varying Dynamics / Mid Freq. Variations	-0.49	0.52	1.07	0.69	0.20
Cluster 3: Varying Timbres / non-iso. Bass	-0.04	-0.31	-0.58	-0.18	0.62
Cluster 4: High Freq. Variations / non-iso. Bass	1.81	-0.86	-0.45	-1.67	0.33

In comparison to cluster 1 and cluster 2, the clusters 3 and 4 score highest with the ‘timbre descriptors’ factor an indicator for fluctuations in the timbre. Furthermore, both clusters show a negative weighting of the ‘low frequency band rhythmical descriptors’ factor II. This can be interpreted as an inversion of the negative IOILowSD factor load, pointing now towards irregular bass events. Cluster 4 is clearly centered around factor I (‘high frequency band rhythmical descriptors’) which is characteristic for rhythmic variations in the upper frequency range (800–5512.5 Hz) of the audio signal.

**Fig 2:**
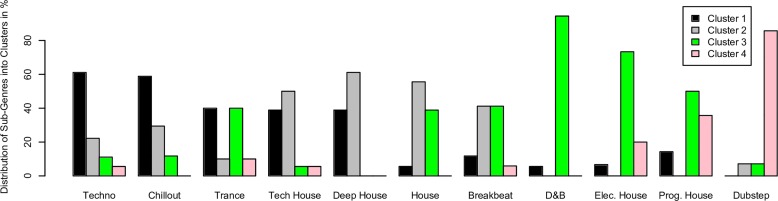
Distribution of the musical stimuli. (A) Musical stimuli are drawn from 11 sub-genres of EDM across the 4 clusters derived from the audio descriptors.

Fig 2 provides frequency counts of cluster memberships, where each musical sample was given a cluster assignment. The distribution for each of the 11 sub-genres to the clusters is provided. Cluster 1 comprises most of the ‘Techno’ and ‘Chill Out’ subgenres, whereas cluster 2 is primarily associated with ‘House’, ‘Deep House’ and ‘Tech House’. The third cluster is associated with the majority of ‘Drum and Bass’, ‘Progressive House’ and ‘Electro House’ samples, whereas the fourth cluster is predominately assigned to ‘Dubstep’ samples.

In summary, four meaningful clusters of EDM emerged with distinct musical attributes: (a) isochronous bass and static timbres, (b) isochronous bass, fluctuating dynamics and rhythmical variation in mid-frequency ranges, (c) non-isochronous bass and fluctuating timbres, and (d) non-isochronous bass with rhythmical variations in the high frequencies.

### Research Question 3: How Do Rating Scale Items Representing Cognitive, Affective, and Psychomotor Domains Vary in Ratings for the Clusters of Musical Stimuli?

#### Summary Statistics

Rasch analyses were conducted using the FACETS [73] computer program. Summary statistics for the MFR-RS Model are found in Table 3. The model analysis indicated overall significance for musical samples (χ^2^_(176)_ = 1582.10, p < .01), raters (χ^2^_(98)_ = 1036.30, p < .01), subgenres (χ^2^_(10)_ = 126.10, p < .01), cluster (χ^2^_(3)_ = 9.40, p = .02), music preference (χ^2^_(4)_ = 51.80, p < .01), and items (χ^2^_(18)_ = 1345.80, p < .01). The reliability of separation for musical samples (REL_musicalsamples_ = 0.89) can be interpreted similarly to traditional test reliability (Cronbach’s Alpha). More specifically, it refers to the reproducibility of relative measure location. Reliability of separation for raters (REL_raters_ = 0.91), clusters (REL_clusters_ = 0.67), subgenres (REL_subgenres_ = 0.91) and items representing the cognitive, affective and psychomotor responses (REL_Items_ = 0.99) is the ratio of true measure variance to observed measure variance. It can be substantively interpreted as the hierarchy of classification evidenced by the spread of each individual rater, each individual musical stimulus, each individual rating scale item, and each individual musical cluster within each respective facet (a facet can be conceptualized as a variable). Separation statistics for rater, item, and subgenre demonstrate a sufficiently wide range of measures. The separation statistic for the clustering, although not unproductive or degrading, implies that the scale may not distinguish as clearly between the high and low clusters [74]. This is most likely due to the small number of clusters (n = 4) and the collapsing of significantly more subgenres (N = 11) into fewer clusters (N = 4). This is to be expected as the analysis consists of only one genre of music with an analysis consisting of only five acoustical property measures. Although a lower separation of reliability is demonstrated for the clusters, REL_clusters_ 0.67 is still considered productive for measurement [75].

**Table 3:** Summary Statistics from the MFR-RS Model showing overall significance for all 6 facets (Musical Sample, Rater, Items, Subgenres, Musical Preference and Clusters derived from the Audio Analysis).

	Facets					
	Musical Sample	Rater	Item	Subgenre	Music Pref	Cluster
**Measure (Logits)**						
*Mean*	0.07	0.00	0.00	0.09	0.06	.07
*SD*	0.19	0.49	0.54	0.16	0.10	.05
*N*	198	99	19	11	5	4
**Infit *MSE***						
*Mean*	0.99	0.98	0.99	0.99	0.97	1.01
*SD*	0.33	0.35	0.29	0.11	0.09	0.08
**Std. Infit *MSE***						
*Mean*	-0.20	-0.30	-0.50	-0.30	-0.70	-0.10
*SD*	1.70	2.40	4.70	2.20	2.60	2.40
**Outfit *MSE***						
*Mean*	1.00	0.99	1.00	0.99	0.98	1.02
*SD*	0.34	0.34	0.30	0.11	0.09	0.07
**Std. Outfit *MSE***						
*Mean*	-0.10	-0.30	-0.40	-0.10	-0.40	0.20
*SD*	1.70	2.30	4.70	2.20	2.60	2.10
**Separation Statistics**						
*Reliability of Separation*	0.89	0.91	0.99	0.91	0.87	0.67
*Chi-Square*	1582.10*	1036.30*	1345.80*	126.10*	51.80*	9.4**
*Degrees of Freedom*	176	98	18	10	4	3

* *p* < 0.01

** *p* = .02

Overall reasonable infit and outfit mean-square (MSE) ranges provide evidence of good model data. Outfit MSE represents outlier-sensitive fit where under- and over-fit for observations of model variance is detected. In other words, this statistic is sensitive to unexpected observations that are less of a threat to the measurement process. Infit MSE represents inlier-pattern sensitive fit where over- and under-fit for Guttman probabilistic patterns are detected. In other words, this statistics represents idiosyncrasies that are hard to diagnose and remedy and present more of a threat to the measurement process. Expected values for parameter-level mean square fit statistics are to center around 1.00 with an acceptable range of .05 to 1.50 for rating scale surveys. Infit and outfit mean square values below .05 indicate responses too predictable (i.e., muted responses, redundancy, data over-fit to the model). Infit and outfit mean square values above 1.50 indicate responses that are too unpredictable (i.e., noisy responses, data under-fit to the model). Standardized infit and outfit statistics are t-tests, reported as z-scores, that test the hypothesis of perfect model data fit for the predictability of data. The expected score for standardized infit and outfit statistics is 0.00 [75]. Less than 0.00 indicates predictability and above 0.00 indicates lack of predictability. Good overall model data fit is evidenced as it fits the range of reasonable predictability (-1.9 to 1.9). For the purpose of this study, data will be referenced according to infit and outfit mean square statistics.

#### Calibration of the Item Facet

**Fig 3:**
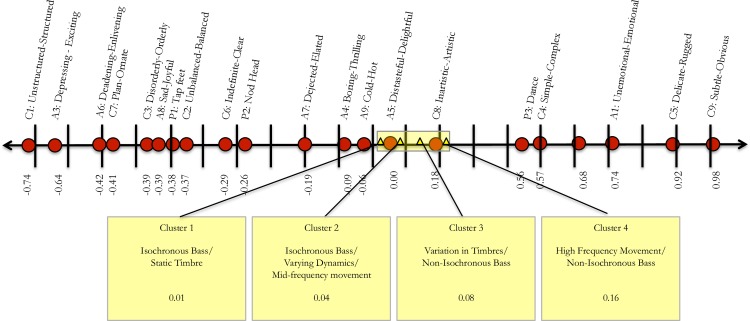
Visual depiction of the cluster calibration in relation to item calibration on the logit scale. (A) Cluster spread ranged from 0.01 logits to 0.16 logits and demonstrated significant distinction according to one fit item: Inartistic/Artistic (C8).

Fig 3 is a visual representation of the spread of the 21 items along the latent continuum. On the right end of the scale in Fig 3 is cognitive item 9 (subtle–obvious), which was the most difficult for raters to endorse as evidenced by a logit location of 0.98. In contrast, on the left end, cognitive item 1 (unstructured–structured) was the easiest item for raters to endorse as evidence by the -0.74 logit location. Table 4 provides the details of the Rasch calibrations of the item facets (i.e., item ratings). To interpret item fit, the threshold of 0.5–1.5 was used to evaluated infit and outfit MSE [76]. Expected values for facet-level mean square fit statistics are to center around 1.00 with an acceptable range of .06 to 1.40 for rating scale surveys [75]. According to these standards, item C9 (subtle–obvious; infit *MSE* = 1.43, outfit *MSE* = 1.57) and item C4 (simple–complex; infit *MSE* = 1.41, outfit *MSE* = 1.40) demonstrated responses too unpredictable for meaningfully measuring raters’ reponses. Items A5 (distasteful–delightful; infit *MSE* = 0.55, outfit *MSE* = 0.56), item A7 (dejected–elated; infit *MSE* = 0.55, outfit *MSE* = 0.56), A8 (sad–joy; infit *MSE* = 0.59, outfit *MSE* = 0.61), and C7 (plain–ornate; infit *MSE* = 0.59, outfit *MSE* = 0.60) demonstrated responses too predictable to be productive for meaningfully measuring raters’ responses. Essentially, items demonstrating under- or over-fit cannot be used in a way that is appropriate nor substantively meaningful for measurement.

**Table 4:** Calibration of the Item Facet. Items (C = cognitive, A = affective, P = psychomotor) are ordered by their endorsability. Most difficult item to rate (C9) on top, easiest (C1) on bottom. C, A, and P items are mixed in their ratings and spread across the logit scale.

Item	Observed Average Rating	Measure	SE	Infit *MSE*	Std. Infit *MSE*	Outfit *MSE*	Std. Outfit *MSE*
C9 (Subtle-Obvious)	2.03	0.98	0.07	1.43	5.80	1.57	7.18
C5 (Delicate-Rugged)	2.07	0.92	0.07	1.16	2.37	1.22	3.06
A1 (Unemotional-Emotional)	2.19	0.74	0.07	1.14	2.14	1.15	2.20
A2 (Unforgettable-Forgettable)	2.23	0.68	0.07	0.91	-1.34	0.95	-0.79
C4 (Simple-Complex)	2.30	0.57	0.06	1.41	5.71	1.40	5.55
P3 (Dance)	2.31	0.56	0.06	1.29	4.21	1.27	3.87
C8 (Inartistic-Artistic)	2.57	0.18	0.06	0.73	-4.58	0.73	-4.60
A5 (Distasteful-Delightful)	2.69	0.00	0.07	0.55	-8.25	0.56	-7.95
A9 (Cold-Hot)	2.73	-0.06	0.07	0.86	-2.16	0.86	-2.14
A4 (Boring-Thrilling)	2.75	-0.09	0.07	0.79	-3.39	0.78	-3.54
A7 (Dejected-Elated)	2.81	-0.19	0.07	0.55	-8.16	0.56	-7.86
P2 (Nod head)	2.86	-0.26	0.07	1.38	5.07	1.34	4.59
C6 (Indefinite-Clear)	2.87	-0.29	0.07	1.12	1.76	1.11	1.55
C2 (Unbalanced-Balanced)	2.93	-0.37	0.07	1.05	0.76	1.06	0.87
P1 (Tap feet)	2.93	-0.38	0.07	1.12	1.69	1.08	1.15
A8 (Sad-Joyful)	2.94	-0.39	0.07	0.59	-7.00	0.61	-6.52
C3 (Disorderly-Orderly)	2.94	-0.39	0.07	1.27	3.68	1.26	3.49
C7 (Plain-Ornate)	2.95	-0.41	0.07	0.59	-6.98	0.60	-6.75
A6 (Deadening-Enlivening)	2.95	-0.42	0.07	0.73	-4.38	0.74	-4.05
A3 (Depressing-Exciting)	3.08	-0.64	0.07	0.78	-3.28	0.76	-3.25
C1 (Unstructured-Structured)	3.14	-0.74	0.07	1.33	4.19	1.33	4.11

*Note*. The items are arranged in measure (endorsability) order, from low to high.

#### Calibration of the Cluster Facet

Fig 3 visually depicts the spread of the four clusters on the latent continuum based upon ratings. Table 5 indicates the empirical calibrations of overall ratings of the four clusters from high to low. Cluster 4 received the highest ratings (0.16 logits) and cluster 1 received the lowest ratings (0.01 logits). Based upon Wright and Linacre’s [75] threshold standards for infit and outfit evaluation, all clusters exhibit good model fit.

**Table 5:** Calibration of the Cluster Facet. Spread of the clusters across the logit scale.

Cluster	Observed Average Rating	Measure	SE	Infit *MSE*	Std. Infit *MSE*	Outfit *MSE*	Std. Outfit *MSE*
Cluster 4 (High Freq. Variations / non-iso. Bass)	2.81	0.16	0.04	1.13	3.17	1.13	2.97
Cluster 3 (Varying Timbres / non-iso. Bass)	2.68	0.08	0.03	0.98	-0.90	0.98	-0.88
Cluster 2 (Isochronous Bass / Varying Dynamics / Mid Freq. Variations)	2.68	0.04	0.03	0.91	-3.40	0.93	-2.66
Cluster 1 (Iso. Bass / Static Timbre)	2.60	0.01	0.03	1.02	0.78	1.04	1.21

In summary, results indicated that affective, cognitive, and psychomotor items were very much mixed in their ratings and ranged from -0.45 logits to 0.98 logits. The calibration of clusters ranged from 0.01 (Cluster 1) to 0.16 logits (Cluster 4), and varied based upon one meaningful item: Item C8 (inartistic–artistic). On average, all clusters were endorsed as being structured (C1), enlivening (A6), ornate (C7), orderly (C3), joyful (A8), conducive to tapping feet (P1), balanced (C2), clear (C6), conducive to nodding head (P2), elated (A7), thrilling (A4), and hot (A9). Cluster 4 (i.e., high frequency variations) was the only cluster to be endorsed as artistic. This means that in order to better make a distinction between clusters 1, 2, and 3, new items with greater precision would have to be added and tested for meaningfulness in measurement. More specifically, all four clusters can be *statistically* differentiated on the logit scale; however, there are not enough items in the same range of the clusters (e.g., 0.01–0.08 logits) to *substantively* differentiate between the clusters using rater responses.

### Research Question 4: Do Meaningful Response Patterns Emerge for Groups of Raters Based upon Musical Preference?

#### Music Preference and Items Representing the Cognitive, Affective and Psychomotor Responses

A differential rater functioning (DRF) analysis was conducted by crossing musical preference with all fit items. Overall differential achievement measures for each item were calculated based upon each item’s standardized rating. The analysis indicated overall statistically significant group differences (χ^2^ = 2196.70, *p* = .02). Pairwise interaction analyses demonstrated four cases (4.76% of total possible interactions) of differential severity and/or leniency. Table 6 provides the interactions for all groups of raters associated with a particular music preference exhibiting differential severity and or leniency (i.e., |Z| +/- 2.0). Raters identifying the sophisticated dimension as their favorite musical dimension (i.e, classical, operatic, avant-garde, world beat, traditional jazz) systemically overestimated musical samples using item C1 (unstructured-structured, *z* = 2.18). On average, observed scores were 132.00, but expected to be 121.81 (a standardized residual of 0.26 logits). Raters identifying the sophisticated dimension as their favorite musical dimension also systematically overestimated musical samples using item C3 (disorderly–orderly, *z* = 2.07). On average, observed scores were 124.00, but expected to be 113.80 (a standardized residual of 0.26 logits). Raters identifying the sophisticated dimension as their favorite musical dimension also systematically underestimated musical samples using item C4 (simple–complex, *z* = -2.54). On average, observed scores were 211.00, but expected to be 233.28 (a standardized residual of -0.22 logits). Lastly, raters identifying the intense dimension as a their favorite musical dimension systematically underestimated musical samples using item P3 (This music makes me want to dance, *z* = -2.68). On average, observed scores were 211.00, but expected to be 233.28 (a standardized residual of -0.22 logits).

**Table 6:** Summary of Differential Facet Functioning Statistics (Music Preference Interactions) for Item Exhibiting | Z | > = 2.0. Showing only selected items, which were significantly overrated by listeners with specific musical preferences.

Music Pref.	Infit MSQ	Outfit MSQ	Item	Total Observed	Total Expected	Stand. Mean Residual (obs-exp)	Bias Logit	*SE*	*Z*
Sophisticated	1.00	1.00	C1 (Unstru-Struct)	132.00	121.81	0.26	0.53	0.22	2.18
Sophisticated	1.30	1.30	C3 (Disord-Ord)	124.00	113.80	0.26	0.46	0.22	2.07
Intense	1.40	1.40	P3 (Dance)	211.00	233.28	-0.22	-0.33	0.12	-2.68
Sophisticated	1.00	0.90	C4 (Simp-Compx)	76.00	89.11	-0.34	-0.52	0.21	-2.54

#### Music Preference and the Clusters of Stimuli

A differential rater functioning (DRF) analysis was conducted by crossing musical preference with all four clusters containing stimuli with certain similarities in their low-level audio descriptors. Overall differential achievement measures for each item were calculated based upon each item’s standardized rating. The analysis indicated overall statistically insignificant group differences (χ^2^ = 13.60, *p* = .85). Therefore, pairwise analyses were not conducted.

In summary, music preference was found to play an important role in some raters’ systematic patterning of ratings in some specific items. In considering scale items, participants that nominated sophisticated music (i.e., classical, operatic, avant-garde, world beat, traditional jazz) as their favorite music found the musical samples to be overly structured (item C1), too orderly (item C3), and too simple (item 4). Additionally, participants that nominated intense music (i.e., classic rock, punk, heavy metal, power pop) as their favorite music found the musical samples to not evoke the urge to dance (item P3).

## Discussion

This study offers a new perspective into the complex relationship between audio events and psychological effects (i.e., positive and negative valence of cognitive, affective, and psychomotor dimensions) of groove-based music by seeking to define a latent, unidimensional construct in a systematic and objective way. In this study we focused on EDM, music specifically produced to be played on the dance floor. However, as no rules or instructions exist how to move the feet, arms or hands to this music, the dance moves to EDM are freely improvised by the listeners and must be encoded into the audio events of the music. To decode the audio events, we extracted low-level audio descriptors and defined clusters of stimuli, which were then mapped to a unifying logit scale, using the Rasch Measurement Model. From the factoring of the audio descriptors we found that stimuli in clusters 1 and 2 were characterized by an isochronous, four-on-the floor bass and less variation in the timbre, in comparison to stimuli in cluster 3 and 4 (variations in timbre, complex rhythmical patterns, non-isochronous bass). The cluster calibration showed that musical samples falling into cluster 3 and 4 were more likely to make listeners want to dance, than it was the case for the tracks falling into cluster 1 and 2. These results support the observations made by Burger [60] who used motion capture systems to evaluate dancer movements and found that variations of the stimulus’ timbre were related to the amount and the speed of head and hand movements in the dances, whereas their feet were mainly stepping to the pulse.

The Rasch analysis provided evidence that all four clusters could be statistically separated on the logit scale. However, the separation was quite narrow (a range of 0.35 logits). Taking into account that all tracks in the pool of stimuli were popular EDM tracks from the year 2014 may explain this cluster distribution, as similar musical features such as timbre, effects, and rhythmic layering often are associated with multiple subgenres of EDM in given time periods [30]. Even the stimuli falling in cluster 1, which received the lowest overall ratings to the cognitive, affective, and psychomotor responses, were indeed popular songs in their specific EDM sub-genres as they too were gleaned from a list of 100 of the most popularly downloaded tracks on the Beatport.com download charts. However, the participants aptly rated these stimuli to have a clear rhythmical structure (Item C6), but perceived these as less 'artistic' (Item C4) than the other stimuli as well as being boring (Item A4) to listen to.

Nevertheless, a clear pulse in the low frequencies allows an easier synchronization of dance moves, also for a larger group of inexperienced dancers [77]. The evolutionary advantage of rhythmically synchronized groups has been discussed in several fields ranging from music education [78] to sport science [79]. Moreover, Phillips-Silver [80] showed that even a subject diagnosed as ‘beat deaf’ was able to dance to the simple rhythm laid down by the strong pulse in the low frequencies characteristic of four-on-the-floor ‘Techno’ music. So why are these tracks less likely to make listeners dance to?

In our case, where the tracks with a non-isochronous bass showed higher ratings towards rhythmical entrainment, it might reflect that the listeners enjoyed if they had to extract the pulse from a more ‘complex’ (C4) metrical structure. This supports the theory of Huron [17] that syncopations and missing downbeats can evoke powerful and positive emotions when listening to music. This is also evidenced as the highest endorsed cluster (cluster 4) being differentiated from the other clusters by the inartistic-artistic item.

Furthermore, the musical preference of the listeners has to be taken into account. In this study we found significant differences between the ratings of participants with different musical preferences. Classical, avant-garde and jazz listeners perceived EDM as too structured, too orderly and too simple, whereas punk, heavy metal and rock listeners were not motivated to dance to EDM although they perceived the music as likely to nod your head to or tap with the feet to it.

From a measurement and scale construction perspective, the raters and clusters could be statistically separated on the logit scale. However, the items used on the scale were only slightly productive in terms of substantive meaning. If the purpose of the measurement is to describe the difference between the clusters based upon items, only one item was meaningful: Item C8 (inartistic–artistic). Furthermore, it was only productive in separating cluster 4 (high frequency variations, non-isochronous bass) from clusters 1, 2, and 3. Therefore, if substantive differentiation between clusters is to be achieved via scale items, the refinement and redevelopment of new items is necessary. The four clusters were all measured above the 13 items marked with the lowest logit measures (items C1, A3, A6, C7, C3, A8, P1, C2, C6, P2, A7, A4 and A9). Although these items were important in describing and contextualizing all of the stimuli, they were not meaningful in differentiating the clusters. Similarly, all four clusters were measured below the 6 items marked with the highest logit measures (items C9, C5, A1, A2, C4, P3). Although these items were important in describing and contextualizing all of the clusters, they too were not meaningful in differentiating the clusters. This is not to say that the individual musical samples were not differentiated by the items, as the individual musical samples ranged from 1.61 logits to -1.63 logits (See S4 Table.). However, with respect to the four clusters, developing new items to target the logit range of 0.01 to 0.36 would provide a substantive differentiation between clusters. Nevertheless, the narrow location of the four clusters on the logit scale might also be an indicator that the extracted low-level audio descriptors were not precise enough to capture all the relevant information in the audio signals. For example, sound design effects like a specific reverb, a unique filter sweep or special settings on the level-compressor for one instrument are characteristic for the EDM genre but are very difficult to extract from a complex audio signal. Using source separation techniques to investigate single instruments might improve the discrimination of the musical stimuli and result in more distinctive cluster solutions.

The same is true from an item perspective. In viewing Fig 3, we see some areas on the line where many items are clustered close together (i.e., items C3, A8, P1, and C2 in the range of -0.39 to -0.37 logits). We also see areas on the line where no items exist (logit location 0.19 through 0.56). If objects of measurement such as clusters, items, or raters fall within the -0.39 to -0.37 logit location, they will be able to be substantively differentiated by those items. However, if objects of measurement fall within the range of 0.19 to 0.56 logits, no substantitive differentiation can be made. In considering the three psychomotor items, there is a considerable logit space between them: Item P1 Tap feet (-0.38 logits), P2 Nod Head (-0.26 logits) and P3 Dance (0.56 logits).

As discussed earlier in this manuscript, the use of Rasch measurement implies the strict requirements of invariant measurement, whereby the items function together conceptually to operationally define a unidimensional construct. In this study, we proposed to operationally define the construct as cognitive, affective, and psychomotor responses. Invariant measurement was achieved in our study as evidenced by overall good model-data fit. Furthermore, the principle of invariant measurement posits that the measurements of the items are not affected by the specific musical samples and the measurements of the musical samples are not affected by the items. Therefore, because good model data fit was observed in the context of this study, we would expect the items to function similarly across different evaluation contexts with different musical samples representing a different musical genre. The question would then remain as to how the items function in relationship between different types of groove- (or even non-groove) based music genres. Overall, the clusters in our study were dispersed in a rather narrow, isolated area of the construct. An interesting follow-up study would be to investigate how different genres of groove-based music (e.g., rock, jazz, funk, etc.) function in relation to the construct. In particular, interest would include how the position of various genres fall in relation to the clusters observed in this study and how their acoustic properties relate to those of the clusters.

A considerable improvement to the scale would be to deeply investigate existing research into specific relationships between body movement and musical structure ([60,81] for instance) and develop a rating scale from the existing literature. A research study that entails the development and validation for such a construct would improvement the measurability of the strength of relationships between musical structure and body movement, both from an experimental and self-reported perspective. Another important followup to this study is the investigation of listeners’ specific methods of listening. A deeper analysis into the individual rater’s specific listening behaviors is warranted. As indicated by Kreutz, Schubert, and Mitchell [82], listening styles can be categorized by two general cognitive styles: empathizing and systemizing. An investigation into the relationship between rater behaviors, rating scale use, and musical stimuli that includes both quantitative and qualitative methodologies may provide a more holistic understanding of listener behavior.

Application of the Rasch measurement with fundamental measurement properties, provides a metric characteristic that is sample-independent [43]. The implication of this is a more consistent connection between research studies. In particular, it can provide an item pool measured using invariant properties that are not limited by the sample (and their individual biases and judgmental idiosyncrasies) evaluating them. In particular, in considering raters’ responses to musical samples, the methodological application of Rasch measurement theory provides a mechanism for calibrating musical samples, items, and raters in a manner that is sample independent [43]. This allows future studies the ability to use one existing scale in a way that can meaningful connect and interpret results. For future studies, the use of this scale in conjunction with musical feature extraction methods and movement feature extraction methods (such as Burger, et. al. [60]) may provide an even more thorough picture of how perceptions of groove-based music and sensorimotor engagement with groove-based music are connected.

## Supporting Information

S1 Table Summary of track information and subgenre labels. (DOCX)

S2 Table 18 sematic differential items from Bartel’s [54] CART-M. (DOCX)

S3 Table The MUSIC Model: Five orthogonal dimensions of music preference [63,64]. (DOCX)

S4 Table Calibration of the musical sample facet. (DOCX)
