# Supplementary material for: There’s More to Groove than Bass in Electronic Dance Music: Why Some People Won’t Dance to Techno
Source: PLoS One. 2016 Oct 31;11(10):e0163938. doi: 10.1371/journal.pone.0163938 (PMC5087899; doi:10.1371/journal.pone.0163938)
Supplement: S1 Table — (DOCX) [file pone.0163938.s001.docx]

**S1 Table. Summary of track information and subgenre labels.**

| **Track ID** | **Artist** | **Track Title** | **Beatport.com Subgenre** |
| --- | --- | --- | --- |
| 1 | The Acid | Ghost (Maya Jane Coles Remix) | Deep House |
| 2 | Metronomy | I'm Acquairus (Edu Imbernon Remix) | Tech House |
| 3 | Mind Against | Strange Days (Recondite Remix) | Techno |
| 4 | NERVO | Ready for the Weekend (Don Diablo) | House |
| 5 | Qulinez | Let's Rock (Original Mix) | Electro house |
| 6 | DallasK | Burn (Original Mix) | Progressive House |
| 7 | Cosmic Gate | Falling Back (Mark Sixma Remix) | Trance |
| 8 | Silent Witness | Being Human (Original Mix) | Drum and Bass |
| 9 | Hannah Wants | Kneadin (Original Mix) | Breakbeat |
| 10 | Diamond Eyes | Oceans (Original mix) | Dubstep |
| 11 | Andrea Roma | Still Loving (Original Mix) | Chillout |
| 12 | Lane 8 | Without You (Original Mix) | Deep House |
| 13 | Sante | All Night Long | Tech House |
| 14 | Mariano Rojo | Trauma (Original Mix) | Techno |
| 15 | Emeskay | Trouble (Original Mix) | House |
| 16 | Mija | Crank It (Original Mix) | Electro house |
| 17 | Julian Jordan | Angels x Demons (Original Mix) | Progressive House |
| 18 | Ram | Clockwork Orange (Original Mix) | Trance |
| 19 | Mefjus | Surrounded (Original Mix) | Drum and Bass |
| 20 | Mafia Kiss | Do It (Original Mix) | Breakbeat |
| 21 | Diamond Eyes | You & Me (Original Mix) | Dubstep |
| 22 | Melosense | Road & Lights (Original Mix) | Chillout |
| 23 | Floating Points | Nectarines (Original Mix) | Deep House |
| 24 | Anja Schneider | Jimmy (Original Mix) | Tech House |
| 25 | Gary Beck | Say What (Adam Beyer Remix) | Techno |
| 26 | JL & Afterman | Black Betty (Original Mix) | House |
| 27 | Danyka Nadeau | Safe & Sound (Original mix) | Electro house |
| 28 | Fox Stevenson | Sweets (Soda Pop) (Original mix) | Progressive House |
| 29 | Susana | Time to Say Goodbye (Original Mix) | Trance |
| 30 | Wickaman | All Jungalist (Original Mix) | Drum and Bass |
| 31 | Wes Smith | Put Ya Hips In It (DJ Fixx remix) | Breakbeat |
| 32 | Knife Party | Give It Up | Dubstep |
| 33 | Soulplace | Her Loving Eyes (Tvardovsky Remix) | Chillout |
| 34 | Ibiza Bass | Benoit & Sergio (Original Mix) | Deep House |
| 35 | Detlef | Forenote (Original Mix) | Tech House |
| 36 | Sasha Carassi | Cube (Original Mix) | Techno |
| 37 | Sidney Charles | Hurricane (Original Mix) | House |
| 38 | Eva Shaw | Space Jungle | Electro house |
| 39 | Wasteland | The Line (Originl Mix) | Progressive House |
| 40 | Omnia | Two Hands (Original Mix) | Trance |
| 41 | Maztek | Sprocket (Original Mix) | Drum and Bass |
| 42 | Zomboy | Patient Zero (Original Mix) | Breakbeat |
| 43 | Bassnectar | You & Me (Original Mix) | Dubstep |
| 44 | 80s Casual | Fiesta (Original Mix) | Chillout |
| 45 | Sailor and I | Turn Around (Ame Remix) | Deep House |
| 46 | Sven Vath | L'esperanza (Ame Reinterpretion) | Tech House |
| 47 | Alan Fitzpatrick | Organic (Original Mix) | Techno |
| 48 | Sandy Rivera | BANG! (EDX's Ibiza Sunrise Remix) | House |
| 49 | Felguk | C'mon Rave On (Original Mix) | Electro house |
| 50 | First State | Get Low (Original Mix) | Progressive House |
| 51 | Armin Van Buuren | Hystereo (Wach Remix) | Trance |
| 52 | Boston | Go With Me (Original Mix) | Drum and Bass |
| 53 | Final Conflict | Shake Ur Body (Original Mix) | Breakbeat |
| 54 | Tritonal | Colors (Culture Code Remix) | Dubstep |
| 55 | Rival Consoles | Haunt (Original Mix) | Chillout |
| 56 | Nora En Pure | Satisfy (Original Mix) | Deep House |
| 57 | Booka Shade | Back to Monza (Original Mix) | Tech House |
| 58 | Agoria | Helice (Original Mix) | Techno |
| 59 | Dario Nunez | Last Mohican (Original Mix) | House |
| 60 | Xilent | The Fall (Original Mix) | Electro house |
| 61 | Wyclef Jean | Divine Sorrow (Original Mix) | Progressive House |
| 62 | Simon Patterson | Whites of Her Eyes (Original Mix) | Trance |
| 63 | Gerra & Stone | Ison (Original Mix) | Drum and Bass |
| 64 | Wes Smith | Everybody Git Down (Original Mix) | Breakbeat |
| 65 | Galantis | You (Brillz Remix) | Dubstep |
| 66 | Thomas Tonfeld | On the Waves(Original Mix) | Chillout |
| 67 | Monte | Bubblegum (Original Mix) | Deep House |
| 68 | Bot | Roof Top (Original Mix) | Tech House |
| 69 | Christian Cambas | Muffin (Original Mix) | Techno |
| 70 | Gallo | Been a Long Time (Dub mix) | House |
| 71 | Joe Garston | Airglow (Middle Milk Remix) | Electro house |
| 72 | Vicetone | United We Dance (Club Mix) | Progressive House |
| 73 | Mike Shiver | Priceless (Original Mix) | Trance |
| 74 | Roni Size | It's a Jazz Thing (Juiceman Remix) | Drum and Bass |
| 75 | Bombo Rosa | Birds (Original Mix) | Breakbeat |
| 76 | Virtual Riot | We're Not Alone VIP (Original mix) | Dubstep |
| 77 | Kasper Bjorke | Heaven (Prins Thomas Mix) | Chillout |
| 78 | London Grammar | Sights (Dennis Ferrer Remix) | Deep House |
| 79 | Sous Sol | Nosotros (Rhadow Remix) | Tech House |
| 80 | Traumer | Hoodium (Original mix) | Techno |
| 81 | Phillip George | Wish You Were Here | House |
| 82 | Joel Fletcher | Bounce Baby (Original Mix) | Electro house |
| 83 | Au Revoire | Rise Early Morning (Extended Mix) | Progressive House |
| 84 | Orkidea | Purity (Sneijder Remix) | Trance |
| 85 | Alix Perez | Modus Calyx & Teebee Remix | Drum and Bass |
| 86 | Nixon | The Basics of Love | Breakbeat |
| 87 | Anna Lunce | Bass Drum Dealer (AC Slater's Remix) | Dubstep |
| 88 | Poncho Warwick | Tainted Jazz | Chillout |
| 89 | Genius of Time | Juno Jam | Deep House |
| 90 | Alex Nigemann | Materium (Original mix) | Tech House |
| 91 | Matador (IE) | Almost Famous (Original Mix) | Techno |
| 92 | Sick Individuals | Wasting Moonlight (L & S Remix) | House |
| 93 | Kiesza | Take U There(TJR Remix) | Electro house |
| 94 | Thomas Newson | Bells at Midnight (Original Mix) | Progressive House |
| 95 | Markus Schultz | Winter Kills Me (F & H Remix) | Trance |
| 96 | Sub Focus | Close (Ivy Lab Remix) | Drum and Bass |
| 97 | Davip | Puncture Wound | Breakbeat |
| 98 | Mat Zo | Ruffneck Bad Boy | Dubstep |
| 99 | PANG! | Lion (Original Mix) | Chillout |
| 100 | Leon Vyehall | It's Just (House of Dupree) | Deep House |
| 101 | Robbie Rivera | The Ride (Original Mix) | Tech House |
| 102 | Johannes Heil | Souls (Original Mix) | Techno |
| 103 | Oliver Heldens | Pikachu (Original Mix) | House |
| 104 | Wiwek | Ground Shake (Original Mix) | Electro house |
| 105 | Twice | X (Original Mix) | Progressive House |
| 106 | Armin Van Buuren | Communication (Paul Oakenfold Mix) | Trance |
| 107 | Mefjus | Saturate (Original Mix) | Drum and Bass |
| 108 | DJ Fixx | Bounce Dat (Original Mix) | Breakbeat |
| 109 | Unnatural Forces | Rick Riddim (Original Mix) | Dubstep |
| 110 | Moosefly | Something in the Trees (Original Mix) | Chillout |
| 111 | Dilby | Tears (Original Mix) | Deep House |
| 112 | Julien Chaptal | Rusty Bicyclette | Tech House |
| 113 | Caribou | Your Love Will Set You Free (C2) | Techno |
| 114 | Chocolate Puma | I Can't Understand (Original Mix) | House |
| 115 | Calvin Harris | Burnin' (Original Mix) | Electro house |
| 116 | Kaskade | A Little More (Extended Mix) | Progressive House |
| 117 | Cold Blue | Furia (Original Mix) | Trance |
| 118 | Rhythm Riders | Give Me a Sign (Bladerunner Remix) | Drum and Bass |
| 119 | Mafia Kiss | Move My Soul (Original Mix) | Breakbeat |
| 120 | Clinton Sly | Lion (Eliminate Remix) | Dubstep |
| 121 | Ben Watt | Bright Star (Sunset Mix) | Chillout |
| 122 | Stereo MC's | Place (Original Mix) | Deep House |
| 123 | Carlos Sanchez | K15 (Detlef Remix) | Tech House |
| 124 | Dimitri Nakov | Don't Stop | Techno |
| 125 | The Magician | Sunlight (Extended Club Mix) | House |
| 126 | R3hab | Karate (Original Mix) | Electro house |
| 127 | Dave Silcox | Shut it Down (Original Mix) | Progressive House |
| 128 | Hristian Hristov | The Beginning (Original Mix) | Trance |
| 129 | Kyza | Countdown (Original Remix) | Drum and Bass |
| 130 | UFO Project | Run that Trap (Original Mix) | Breakbeat |
| 131 | Dubloadz | Fight Music (VIP) | Dubstep |
| 132 | Tipper | No Dice | Chillout |
| 133 | Watermat | Bullilt | Deep House |
| 134 | Patrick Topping | Forget (Original Mix) | Tech House |
| 135 | Marc Romboy | Iceland (Laurent Garnier Remix) | Techno |
| 136 | Romanthony | Testify (Jimmy Edgar Edit) | House |
| 137 | MUST DIE! | Hellcat (Habstrakt Remix) | Electro house |
| 138 | Quintino | Slammer (Original Mix) | Progressive House |
| 139 | Max Freegrant | Final Race (Original Mix) | Trance |
| 140 | Andy C | Heartbeat Loud (Extended Version) | Drum and Bass |
| 141 | Kuplay | Homeboy (Original Mix) | Breakbeat |
| 142 | Luciana | Night Shine (Original Mix) | Dubstep |
| 143 | Hard Ton | Walking in the Night (Original Mix) | Chillout |
| 144 | Johannes Brecht | Another World (Original Mix) | Deep House |
| 145 | Joeski | It's all the Same | Tech House |
| 146 | Guy J | Candyland (King Unique Remix) | Techno |
| 147 | Ed Ed | I Got (Oliver Dollar Remix) | House |
| 148 | Freaky Bass | Floss (Original Mix) | Electro house |
| 149 | Alesso | Heros (we could be) (Extended Mix) | Progressive House |
| 150 | Jordan Suckley | Elation (Joint Operations Centre Remix) | Trance |
| 151 | Black Sun Empire | Ego (Original Mix) | Drum and Bass |
| 152 | Noisestorm | Sentinel (Original Mix) | Breakbeat |
| 153 | 501 | Crystallize (Original Mix) | Dubstep |
| 154 | Can 7 | Uska Dara (Original Mix) | Chillout |
| 155 | Maya Jane Coles | I Would Fly (Original Mix) | Deep House |
| 156 | Wouter S | Stutter (Original Mix) | Tech House |
| 157 | Eduardo Calle | The Demigod's Control (Original Mix) | Techno |
| 158 | Nightcrawlers | Push the Feeling On (JJ Remix) | House |
| 159 | Tom Swoon | Ghost (Original Mix) | Electro house |
| 160 | Coldplay | A Sky Full of Stars (Hardwell Remix) | Progressive House |
| 161 | John O'Callaghan | The Saw (Original Mix) | Trance |
| 162 | Tantrum Desire | Genesis (Original Mix) | Drum and Bass |
| 163 | DJ Fixx | We Run This (Original Mix) | Breakbeat |
| 164 | The Frim | Hi (Original Mix) | Dubstep |
| 165 | Crazy P | Heartbreaker | Chillout |
| 166 | Ten Walls | Requiem (Original Mix) | Deep House |
| 167 | Clause VonStroke | LFO I Love You | Tech House |
| 168 | 2000 and One | Plant 1 (Original Mix) | Techno |
| 169 | Italobros | Just Take (Original Mix) | House |
| 170 | Tony Junior | Jump Around (Original Mix) | Electro house |
| 171 | Kolsch | All That Matters (Kryder Remix) | Progressive House |
| 172 | Gareth Emery | U (Bryan Kearney Remix) | Trance |
| 173 | Alix Perez | Dark Days (Fourward Remix) | Drum and Bass |
| 174 | Maztek | Bad Body (Original Mix) | Breakbeat |
| 175 | Getter | Dubstep is Dead (Original Mix) | Dubstep |
| 176 | Gramatik | Sumthin' (Original Mix) | Chillout |
| 177 | Teenage Mutants | Horn (Original Mix) | Deep House |
| 178 | Mason | Exceeder (UMEK & Mike Vale Remix) | Tech House |
| 179 | Diction | Suits (Drumcomplex Remix) | Techno |
| 180 | Oliver Heldens | Koala (Original mix) | House |
| 181 | Bassjackers | X (Original Mix) | Electro house |
| 182 | Merk & Kremont | Now or Never (Original Mix) | Progressive House |
| 183 | Nevv Kenedy | Be the Light (Original Mix) | Trance |
| 184 | Jubei | These Things VIP (Original Mix) | Drum and Bass |
| 185 | Keith Mackenzie | 4 Ur Luv (Original Mix) | Breakbeat |
| 186 | Tim Ismag | Anime Fight | Dubstep |
| 187 | Cloud Boat | Lions on the Beach | Chillout |
| 188 | Howson's Groove | Can't Explain (Celsius Remix) | Deep House |
| 189 | Metronomy | Love Letters (Agoria Remix) | Tech House |
| 190 | Johannes Heil | Hectic (Original Mix) | Techno |
| 191 | Oliver Dollar | Pushing On (Tchami Remix) | House |
| 192 | Ummet Ozcan | Overdrive (Part 2) (Club Edit) | Electro house |
| 193 | Mark Sixma | Rise Up (Original Mix) | Progressive House |
| 194 | Mr. Pit | Exposure (Original Mix) | Trance |
| 195 | Culture Shock | Troglodyte VIP (Original Mix) | Drum and Bass |
| 196 | Deekline | Sound of Music (Deekline Bass Mix) | Breakbeat |
| 197 | Velvetine | The Great Divide (Seven Lions Remix) | Dubstep |
| 198 | Joachim Pastor | Kenia (Original Mix) | Chillout |
