# Supplementary material for: There’s More to Groove than Bass in Electronic Dance Music: Why Some People Won’t Dance to Techno
Source: PLoS One. 2016 Oct 31;11(10):e0163938. doi: 10.1371/journal.pone.0163938 (PMC5087899; doi:10.1371/journal.pone.0163938)
Supplement: S2 Table — (DOCX) [file pone.0163938.s002.docx]

**S2 Table.** **18 sematic differential items from Bartel’s [54] CART-M.**

| 1. | Exciting/depressing |
| --- | --- |
| 2. | Enlivening/deadening |
| 3. | Thrilling/boring |
| 4. | Elated/dejected |
| 5. | Joyful/sad |
| 6. | Delightful/distasteful |
| 7. | Emotional/unemotional |
| 8. | Hot/cold |
| 9. | Unforgettable/forgettable |
| 10. | Structured/unstructured |
| 11. | Orderly/disorderly |
| 12. | Balanced/unbalanced |
| 13. | Artistic/inartistic |
| 14. | Clear/indefinite |
| 15. | Complex/simple |
| 16. | Ornate/plain |
| 17. | Subtle/obvious |
| 18. | Delicate/rugged |
