# Supplementary material for: There’s More to Groove than Bass in Electronic Dance Music: Why Some People Won’t Dance to Techno
Source: PLoS One. 2016 Oct 31;11(10):e0163938. doi: 10.1371/journal.pone.0163938 (PMC5087899; doi:10.1371/journal.pone.0163938)
Supplement: S3 Table — (DOCX) [file pone.0163938.s003.docx]

**S3 Table.** **The MUSIC Model: Five orthogonal dimensions of music preference [63,64].**

| **Dimension** | **Description** | **Examples** |
| --- | --- | --- |
| Mellow | Romantic, relaxing, unaggressive, sad, slow, quiet | soft rock, R & B, adult contemporary |
| Unpretentious | Uncomplicated, relaxing, unaggressive, soft, acoustic | country, folk, singer/songwriter |
| Sophisticated | Inspiring, intelligent, complex, dynamic | classical, operatic, avant-garde, world beat, traditional jazz |
| Intense | Distorted, loud, aggressive, not relaxing, not romantic | Classic rock, punk, heavy metal, power pop |
| Contemporary | Percussive, electric, not sad | Rap, electronica, Latin, acid jazz, Euro pop |
