# Supplementary material for: There’s More to Groove than Bass in Electronic Dance Music: Why Some People Won’t Dance to Techno
Source: PLoS One. 2016 Oct 31;11(10):e0163938. doi: 10.1371/journal.pone.0163938 (PMC5087899; doi:10.1371/journal.pone.0163938)
Supplement: S4 Table — (DOCX) [file pone.0163938.s004.docx]

| S4 Table. Calibration of the musical sample facet. | | | | | | | |
| --- | --- | --- | --- | --- | --- | --- | --- |
| Musical Sample | Observed Average Rating | Measure | SE | Infit *MSE* | Std. Infit *MSE* | Outfit *MSE* | Std. Outfit *MSE* |
|  |  |  |  |  |  |  |  |
| 54 | 3.40 | 1.61 | 0.23 | 1.29 | 1.23 | 1.28 | 1.17 |
| 107 | 2.93 | 1.56 | 0.20 | 1.08 | 0.46 | 1.05 | 0.32 |
| 94 | 3.33 | 1.31 | 0.23 | 0.44 | -3.07 | 0.44 | -2.77 |
| 143 | 3.07 | 1.24 | 0.20 | 1.87 | 3.49 | 1.79 | 3.17 |
| 110 | 3.17 | 1.19 | 0.21 | 0.84 | -0.75 | 0.87 | -0.55 |
| 17 | 3.10 | 1.16 | 0.20 | 1.18 | 0.90 | 1.24 | 1.13 |
| 194 | 3.14 | 1.13 | 0.21 | 0.76 | -1.23 | 0.80 | -0.95 |
| 180 | 3.21 | 1.10 | 0.21 | 1.11 | 0.57 | 0.98 | -0.03 |
| 162 | 2.74 | 1.01 | 0.19 | 0.63 | -2.11 | 0.63 | -2.07 |
| 172 | 3.14 | 1.00 | 0.21 | 0.82 | -0.83 | 0.86 | -0.65 |
| 108 | 2.76 | 0.99 | 0.19 | 0.64 | -2.14 | 0.65 | -2.05 |
| 141 | 3.17 | 0.99 | 0.21 | 1.54 | 2.25 | 1.37 | 1.57 |
| 121 | 3.26 | 0.95 | 0.22 | 0.82 | -0.81 | 0.83 | -0.75 |
| 142 | 3.19 | 0.94 | 0.21 | 1.54 | 2.24 | 1.38 | 1.60 |
| 196 | 3.21 | 0.90 | 0.21 | 0.90 | -0.40 | 0.81 | -0.84 |
| 64 | 3.29 | 0.89 | 0.22 | 0.73 | -1.30 | 0.68 | -1.56 |
| 80 | 2.95 | 0.89 | 0.20 | 0.33 | -4.69 | 0.35 | -4.40 |
| 170 | 3.07 | 0.87 | 0.20 | 0.97 | -0.10 | 1.07 | 0.38 |
| 160 | 2.90 | 0.85 | 0.20 | 0.78 | -1.16 | 0.79 | -1.03 |
| 92 | 3.43 | 0.84 | 0.23 | 1.21 | 0.94 | 1.03 | 0.19 |
| 14 | 2.98 | 0.83 | 0.20 | 0.57 | -2.56 | 0.58 | -2.46 |
| 158 | 3.12 | 0.78 | 0.20 | 0.38 | -3.97 | 0.40 | -3.76 |
| 159 | 2.76 | 0.78 | 0.19 | 0.60 | -2.35 | 0.63 | -2.10 |
| 32 | 3.00 | 0.77 | 0.20 | 1.16 | 0.83 | 1.12 | 0.63 |
| 177 | 2.93 | 0.77 | 0.19 | 0.82 | -0.89 | 0.79 | -1.10 |
| 157 | 2.83 | 0.76 | 0.19 | 0.60 | -2.36 | 0.62 | -2.21 |
| 198 | 3.19 | 0.75 | 0.21 | 1.68 | 2.78 | 1.60 | 2.45 |
| 90 | 2.88 | 0.68 | 0.20 | 0.87 | -0.60 | 0.85 | -0.71 |
| 91 | 3.14 | 0.68 | 0.21 | 1.66 | 2.74 | 1.67 | 2.75 |
| 3 | 2.86 | 0.66 | 0.19 | 1.21 | 1.09 | 1.22 | 1.13 |
| 40 | 2.95 | 0.66 | 0.21 | 1.08 | 0.45 | 1.01 | 0.14 |
| 43 | 3.00 | 0.66 | 0.20 | 0.49 | -3.09 | 0.51 | -2.95 |
| 15 | 2.88 | 0.64 | 0.19 | 0.77 | -1.25 | 0.75 | -1.36 |
| 56 | 2.76 | 0.64 | 0.19 | 0.84 | -0.81 | 0.87 | -0.66 |
| 19 | 2.62 | 0.60 | 0.19 | 0.65 | -2.04 | 0.64 | -2.09 |
| 72 | 3.21 | 0.55 | 0.21 | 0.65 | -1.84 | 0.64 | -1.81 |
| 169 | 2.88 | 0.55 | 0.19 | 0.85 | -0.75 | 0.94 | -0.28 |
| 79 | 2.81 | 0.52 | 0.19 | 0.62 | -2.26 | 0.62 | -2.22 |
| 149 | 3.33 | 0.52 | 0.22 | 0.97 | -0.07 | 0.88 | -0.49 |
| 191 | 3.07 | 0.51 | 0.20 | 0.91 | -0.36 | 0.87 | -0.56 |
| 71 | 3.10 | 0.49 | 0.20 | 0.68 | -1.69 | 0.72 | -1.45 |
| 4 | 3.05 | 0.48 | 0.20 | 1.33 | 1.58 | 1.24 | 1.15 |
| 13 | 2.86 | 0.46 | 0.19 | 0.85 | -0.76 | 0.84 | -0.81 |
| 53 | 2.67 | 0.46 | 0.19 | 0.55 | -2.84 | 0.55 | -2.85 |
| 93 | 2.79 | 0.45 | 0.20 | 0.96 | -0.16 | 0.94 | -0.25 |
| 45 | 2.71 | 0.43 | 0.19 | 0.55 | -2.80 | 0.56 | -2.71 |
| 12 | 2.88 | 0.41 | 0.19 | 1.04 | 0.29 | 1.01 | 0.12 |
| 119 | 3.10 | 0.39 | 0.20 | 1.11 | 0.57 | 1.06 | 0.34 |
| 77 | 3.02 | 0.37 | 0.20 | 0.60 | -2.27 | 0.58 | -2.40 |
| 87 | 2.76 | 0.37 | 0.19 | 0.65 | -2.03 | 0.65 | -2.02 |
| 105 | 2.67 | 0.37 | 0.20 | 0.95 | -0.20 | 0.94 | -0.23 |
| 137 | 2.83 | 0.37 | 0.19 | 1.20 | 1.04 | 1.16 | 0.82 |
| 138 | 2.98 | 0.36 | 0.20 | 1.05 | 0.30 | 1.03 | 0.20 |
| 146 | 2.62 | 0.36 | 0.19 | 1.54 | 2.52 | 1.58 | 2.68 |
| 31 | 2.64 | 0.35 | 0.19 | 0.85 | -0.80 | 0.84 | -0.85 |
| 33 | 2.67 | 0.35 | 0.19 | 1.04 | 0.27 | 1.05 | 0.34 |
| 41 | 2.21 | 0.33 | 0.19 | 0.92 | -0.39 | 0.90 | -0.47 |
| 171 | 2.83 | 0.33 | 0.19 | 1.30 | 1.51 | 1.27 | 1.34 |
| 189 | 2.90 | 0.32 | 0.19 | 0.87 | -0.65 | 0.85 | -0.72 |
| 52 | 2.55 | 0.31 | 0.19 | 0.71 | -1.67 | 0.72 | -1.63 |
| 98 | 3.14 | 0.31 | 0.21 | 1.18 | 0.91 | 1.21 | 1.00 |
| 109 | 2.81 | 0.31 | 0.19 | 1.09 | 0.53 | 1.11 | 0.59 |
| 156 | 2.57 | 0.31 | 0.19 | 0.89 | -0.54 | 0.88 | -0.58 |
| 34 | 2.55 | 0.29 | 0.19 | 0.64 | -2.13 | 0.68 | -1.83 |
| 50 | 3.24 | 0.28 | 0.22 | 1.09 | 0.47 | 1.02 | 0.15 |
| 89 | 2.64 | 0.28 | 0.19 | 1.43 | 2.03 | 1.38 | 1.83 |
| 11 | 2.83 | 0.25 | 0.19 | 1.31 | 1.53 | 1.32 | 1.59 |
| 133 | 2.90 | 0.25 | 0.20 | 0.89 | -0.50 | 0.86 | -0.67 |
| 135 | 2.67 | 0.25 | 0.19 | 1.16 | 0.85 | 1.14 | 0.78 |
| 16 | 2.62 | 0.23 | 0.19 | 1.26 | 1.35 | 1.26 | 1.33 |
| 27 | 3.31 | 0.23 | 0.22 | 1.25 | 1.10 | 1.44 | 1.79 |
| 118 | 2.86 | 0.23 | 0.19 | 0.33 | -4.73 | 0.31 | -4.87 |
| 1 | 2.69 | 0.21 | 0.19 | 1.27 | 1.40 | 1.26 | 1.34 |
| 81 | 3.07 | 0.21 | 0.21 | 0.64 | -1.90 | 0.95 | -0.15 |
| 112 | 2.60 | 0.21 | 0.19 | 0.81 | -1.04 | 0.83 | -0.89 |
| 20 | 2.52 | 0.18 | 0.19 | 0.80 | -1.06 | 0.79 | -1.13 |
| 24 | 2.83 | 0.18 | 0.19 | 0.88 | -0.60 | 0.86 | -0.70 |
| 95 | 2.48 | 0.18 | 0.19 | 0.64 | -2.09 | 0.67 | -1.85 |
| 125 | 3.19 | 0.18 | 0.21 | 0.81 | -0.88 | 0.77 | -1.11 |
| 197 | 3.02 | 0.18 | 0.20 | 1.13 | 0.68 | 1.12 | 0.62 |
| 165 | 3.00 | 0.17 | 0.20 | 0.83 | -0.82 | 0.87 | -0.62 |
| 167 | 2.71 | 0.17 | 0.19 | 0.59 | -2.48 | 0.59 | -2.48 |
| 74 | 2.71 | 0.16 | 0.19 | 0.66 | -1.93 | 0.65 | -2.01 |
| 67 | 2.69 | 0.15 | 0.19 | 0.51 | -3.14 | 0.52 | -3.00 |
| 78 | 2.81 | 0.15 | 0.19 | 0.73 | -1.48 | 0.72 | -1.53 |
| 123 | 2.69 | 0.14 | 0.19 | 0.92 | -0.36 | 0.92 | -0.40 |
| 101 | 2.88 | 0.12 | 0.19 | 0.79 | -1.08 | 0.76 | -1.28 |
| 154 | 2.76 | 0.07 | 0.19 | 0.92 | -0.36 | 0.90 | -0.48 |
| 130 | 3.17 | 0.06 | 0.21 | 0.95 | -0.14 | 0.86 | -0.61 |
| 36 | 2.76 | 0.05 | 0.19 | 0.67 | -1.94 | 0.68 | -1.83 |
| 193 | 2.64 | 0.05 | 0.19 | 0.45 | -3.71 | 0.44 | -3.75 |
| 155 | 2.40 | 0.04 | 0.18 | 1.36 | 1.82 | 1.40 | 1.99 |
| 63 | 2.52 | 0.03 | 0.19 | 0.78 | -1.23 | 0.79 | -1.17 |
| 51 | 2.48 | 0.02 | 0.19 | 0.91 | -0.44 | 0.90 | -0.50 |
| 117 | 2.88 | 0.02 | 0.19 | 0.93 | -0.30 | 0.91 | -0.41 |
| 120 | 2.95 | 0.02 | 0.20 | 1.46 | 2.12 | 1.41 | 1.89 |
| 21 | 2.86 | 0.01 | 0.19 | 1.38 | 1.83 | 1.37 | 1.79 |
| 103 | 2.95 | 0.01 | 0.20 | 0.75 | -1.35 | 0.73 | -1.44 |
| 122 | 2.62 | 0.01 | 0.19 | 0.84 | -0.85 | 0.84 | -0.81 |
| 9 | 2.95 | 0.00 | 0.20 | 1.13 | 0.70 | 1.17 | 0.88 |
| 85 | 2.64 | 0.00 | 0.19 | 0.74 | -1.45 | 0.74 | -1.44 |
| 134 | 2.74 | 0.00 | 0.20 | 0.82 | -0.93 | 0.87 | -0.60 |
| 173 | 2.21 | 0.00 | 0.19 | 0.66 | -2.00 | 0.68 | -1.86 |
| 2 | 2.52 | -0.01 | 0.19 | 1.18 | 0.97 | 1.18 | 0.96 |
| 124 | 2.50 | -0.03 | 0.19 | 0.98 | -0.05 | 1.02 | 0.18 |
| 145 | 2.43 | -0.03 | 0.19 | 1.04 | 0.30 | 1.08 | 0.46 |
| 65 | 2.69 | -0.07 | 0.19 | 1.61 | 2.79 | 1.61 | 2.79 |
| 175 | 2.45 | -0.09 | 0.19 | 0.96 | -0.16 | 0.93 | -0.33 |
| 5 | 2.52 | -0.13 | 0.19 | 1.18 | 0.96 | 1.18 | 0.96 |
| 163 | 2.71 | -0.13 | 0.19 | 0.95 | -0.22 | 0.93 | -0.31 |
| 102 | 2.64 | -0.15 | 0.19 | 1.06 | 0.40 | 1.09 | 0.52 |
| 8 | 2.57 | -0.18 | 0.19 | 1.37 | 1.82 | 1.39 | 1.89 |
| 42 | 2.07 | -0.20 | 0.19 | 0.78 | -1.21 | 0.77 | -1.19 |
| 55 | 2.24 | -0.22 | 0.19 | 1.22 | 1.18 | 1.18 | 0.96 |
| 168 | 2.36 | -0.22 | 0.19 | 0.80 | -1.10 | 0.78 | -1.22 |
| 140 | 2.26 | -0.23 | 0.19 | 1.31 | 1.56 | 1.51 | 2.36 |
| 176 | 2.17 | -0.23 | 0.19 | 1.13 | 0.72 | 1.29 | 1.44 |
| 6 | 2.62 | -0.24 | 0.19 | 1.15 | 0.84 | 1.15 | 0.81 |
| 82 | 2.81 | -0.24 | 0.20 | 0.78 | -1.15 | 0.81 | -0.95 |
| 126 | 2.93 | -0.26 | 0.20 | 0.86 | -0.65 | 0.84 | -0.78 |
| 115 | 2.86 | -0.27 | 0.19 | 0.99 | 0.02 | 0.98 | -0.02 |
| 114 | 2.62 | -0.28 | 0.19 | 1.21 | 1.12 | 1.27 | 1.36 |
| 178 | 2.19 | -0.29 | 0.19 | 1.00 | 0.06 | 1.10 | 0.57 |
| 147 | 2.67 | -0.30 | 0.19 | 0.75 | -1.37 | 0.74 | -1.44 |
| 75 | 2.79 | -0.32 | 0.19 | 0.61 | -2.31 | 0.63 | -2.15 |
| 96 | 2.02 | -0.32 | 0.20 | 1.19 | 0.97 | 1.21 | 1.02 |
| 70 | 2.74 | -0.33 | 0.19 | 0.52 | -3.07 | 0.51 | -3.10 |
| 69 | 2.45 | -0.36 | 0.18 | 0.62 | -2.30 | 0.64 | -2.2 |
| 148 | 2.57 | -0.37 | 0.19 | 1.13 | 0.74 | 1.13 | 0.73 |
| 185 | 2.43 | -0.37 | 0.19 | 1.06 | 0.40 | 1.04 | 0.27 |
| 25 | 2.52 | -0.38 | 0.19 | 1.05 | 0.35 | 1.06 | 0.37 |
| 104 | 2.71 | -0.39 | 0.19 | 1.14 | 0.78 | 1.13 | 0.72 |
| 195 | 2.24 | -0.39 | 0.19 | 1.03 | 0.21 | 1.25 | 1.30 |
| 111 | 2.19 | -0.40 | 0.19 | 1.15 | 0.84 | 1.14 | 0.78 |
| 150 | 2.60 | -0.40 | 0.19 | 0.62 | -2.29 | 0.61 | -2.34 |
| 184 | 2.07 | -0.41 | 0.19 | 0.82 | -0.96 | 0.83 | -0.90 |
| 60 | 2.76 | -0.42 | 0.19 | 1.38 | 1.84 | 1.37 | 1.76 |
| 144 | 1.90 | -0.42 | 0.20 | 1.67 | 2.90 | 1.61 | 2.56 |
| 7 | 2.52 | -0.44 | 0.19 | 1.02 | 0.19 | 1.01 | 0.12 |
| 179 | 1.90 | -0.44 | 0.20 | 1.49 | 2.25 | 1.39 | 1.81 |
| 35 | 2.45 | -0.45 | 0.19 | 0.43 | -3.86 | 0.44 | -3.83 |
| 86 | 2.55 | -0.45 | 0.19 | 1.15 | 0.84 | 1.14 | 0.77 |
| 187 | 2.55 | -0.45 | 0.19 | 1.10 | 0.56 | 1.11 | 0.62 |
| 174 | 2.10 | -0.49 | 0.19 | 2.07 | 4.42 | 2.09 | 4.41 |
| 30 | 2.38 | -0.53 | 0.19 | 1.30 | 1.51 | 1.28 | 1.40 |
| 44 | 2.07 | -0.53 | 0.19 | 0.85 | -0.75 | 0.95 | -0.20 |
| 58 | 2.33 | -0.53 | 0.19 | 1.64 | 2.91 | 1.66 | 2.96 |
| 68 | 2.31 | -0.53 | 0.19 | 0.65 | -2.10 | 0.66 | -1.98 |
| 88 | 2.07 | -0.53 | 0.19 | 1.18 | 0.93 | 1.40 | 1.88 |
| 161 | 1.93 | -0.53 | 0.20 | 0.80 | -1.00 | 0.73 | -1.30 |
| 23 | 2.38 | -0.54 | 0.19 | 1.21 | 1.13 | 1.28 | 1.46 |
| 151 | 2.38 | -0.55 | 0.19 | 0.59 | -2.55 | 0.62 | -2.31 |
| 100 | 2.45 | -0.56 | 0.18 | 1.62 | 2.88 | 1.66 | 3.04 |
| 59 | 2.69 | -0.57 | 0.19 | 2.08 | 4.42 | 2.21 | 4.79 |
| 127 | 3.07 | -0.60 | 0.20 | 1.30 | 1.40 | 1.24 | 1.15 |
| 26 | 2.64 | -0.62 | 0.19 | 0.67 | -1.93 | 0.65 | -2.06 |
| 136 | 2.24 | -0.67 | 0.19 | 0.91 | -0.44 | 0.92 | -0.37 |
| 22 | 2.21 | -0.68 | 0.19 | 1.29 | 1.51 | 1.48 | 2.30 |
| 97 | 2.48 | -0.70 | 0.19 | 1.77 | 3.45 | 1.77 | 3.43 |
| 57 | 2.31 | -0.71 | 0.19 | 0.93 | -0.34 | 0.97 | -0.10 |
| 84 | 2.64 | -0.73 | 0.19 | 0.82 | -0.93 | 0.82 | -0.93 |
| 166 | 2.33 | -0.73 | 0.19 | 0.84 | -0.86 | 0.85 | -0.80 |
| 181 | 2.07 | -0.73 | 0.19 | 1.06 | 0.36 | 1.04 | 0.28 |
| 132 | 2.55 | -0.74 | 0.19 | 1.37 | 1.81 | 1.42 | 2.02 |
| 99 | 2.38 | -0.75 | 0.18 | 1.84 | 3.71 | 1.88 | 3.86 |
| 46 | 1.90 | -0.77 | 0.20 | 1.01 | 0.13 | 0.98 | -0.02 |
| 113 | 1.98 | -0.77 | 0.20 | 0.81 | -0.97 | 0.78 | -1.10 |
| 47 | 2.24 | -0.78 | 0.19 | 1.19 | 0.98 | 1.14 | 0.75 |
| 182 | 2.17 | -0.83 | 0.19 | 1.18 | 0.96 | 1.13 | 0.71 |
| 188 | 2.17 | -0.85 | 0.19 | 0.80 | -1.08 | 0.82 | -0.93 |
| 129 | 2.38 | -0.88 | 0.19 | 1.35 | 1.73 | 1.49 | 2.31 |
| 164 | 2.29 | -0.89 | 0.19 | 0.82 | -0.96 | 0.83 | -0.91 |
| 37 | 2.52 | -0.93 | 0.19 | 0.69 | -1.81 | 0.69 | -1.85 |
| 10 | 2.38 | -1.01 | 0.19 | 1.04 | 0.25 | 1.03 | 0.24 |
| 28 | 2.43 | -1.27 | 0.19 | 0.82 | -0.95 | 0.82 | -0.98 |
| 190 | 1.71 | -1.36 | 0.21 | 1.18 | 0.89 | 1.72 | 2.76 |
| 131 | 2.12 | -1.63 | 0.19 | 1.40 | 1.94 | 1.58 | 2.61 |
